# Endothelin-1, Outcomes in Patients With Heart Failure and Reduced Ejection Fraction, and Effects of Dapagliflozin: Findings From DAPA-HF

**DOI:** 10.1161/CIRCULATIONAHA.122.063327

**Published:** 2023-04-11

**Authors:** Su Ern Yeoh, Kieran F. Docherty, Ross T. Campbell, Pardeep S. Jhund, Ann Hammarstedt, Hiddo J.L. Heerspink, Petr Jarolim, Lars Køber, Mikhail N. Kosiborod, Felipe A. Martinez, Piotr Ponikowski, Scott D. Solomon, Mikaela Sjöstrand, Olof Bengtsson, Peter J. Greasley, Naveed Sattar, Paul Welsh, Marc S. Sabatine, David A. Morrow, John J.V. McMurray

**Affiliations:** 1 British Heart Foundation Cardiovascular Research Centre, University of Glasgow, United Kingdom (S.E.Y., K.F.D., R.T.C., P.S.J., N.S., P.W., J.J.V.M.).; 2 BioPharmaceuticals Research and Development, AstraZeneca, Gothenburg, Sweden (A.H., M.S., O.B., P.J.G.).; 3 Department of Clinical Pharmacy and Pharmacology, University of Groningen, University Medical Center Groningen, The Netherlands (H.J.L.H.).; 4 George Institute for Global Health, University of New South Wales, Sydney, Australia (H.J.L.H.).; 5 Department of Pathology (P.J.), Brigham and Women’s Hospital, Boston, MA.; 6 Thrombolysis in Myocardial Infarction Study Group (M.S.S., D.A.M.), Brigham and Women’s Hospital, Boston, MA.; 7 Division of Cardiovascular Medicine (S.D.S.), Brigham and Women’s Hospital, Boston, MA.; 8 Department of Cardiology, Rigshospitalet, Copenhagen University Hospital, Denmark (L.K.).; 9 Saint Luke’s Mid America Heart Institute, University of Missouri, Kansas City (M.N.K.).; 10 Universidad Nacional de Córdoba, Argentina (F.A.M.).; 11 Center for Heart Diseases, University Hospital, Wroclaw Medical University, Poland (P.P.).

**Keywords:** biomarkers, endothelin-1, heart failure, kidney, SGLT-2 inhibitor

## Abstract

ET-1 (endothelin-1) is implicated in the pathophysiology of heart failure and renal disease. Its prognostic importance and relationship with kidney function in patients with heart failure with reduced ejection fraction receiving contemporary treatment are uncertain. We investigated these and the efficacy of dapagliflozin according to ET-1 level in the DAPA-HF trial (Dapagliflozin and Prevention of Adverse Outcomes in Heart Failure).

**Methods:**

We investigated the incidence of the primary outcome (cardiovascular death or worsening heart failure), change in kidney function, and the effect of dapagliflozin according to baseline ET-1 concentration, adjusting in Cox models for other recognized prognostic variables in heart failure including NT-proBNP (N-terminal pro-B-type natriuretic peptide). We also examined the effect of dapagliflozin on ET-1 level.

**Results:**

Overall, 3048 participants had baseline ET-1 measurements: tertile 1 (T1; ≤3.28 pg/mL; n=1016); T2 (>3.28–4.41 pg/mL; n=1022); and T3 (>4.41 pg/mL; n=1010). Patients with higher ET-1 were more likely male, more likely obese, and had lower left ventricular ejection fraction, lower estimated glomerular filtration rate, worse functional status, and higher NT-proBNP and hs-TnT (high-sensitivity troponin-T). In the adjusted Cox models, higher baseline ET-1 was independently associated with worse outcomes and steeper decline in kidney function (adjusted hazard ratio for primary outcome of 1.95 [95% CI, 1.53–2.50] for T3 and 1.36 [95% CI, 1.06–1.75] for T2; both versus T1; estimated glomerular filtration rate slope: T3, –3.19 [95% CI, –3.66 to –2.72] mL/min per 1.73 m^2^ per y, T2, –2.08 [95% CI, –2.52 to –1.63] and T1 –2.35 [95% CI, –2.79 to –1.91]; *P*=0.002). The benefit of dapagliflozin was consistent regardless of baseline ET-1, and the placebo-corrected decrease in ET-1 with dapagliflozin was 0.13 pg/mL (95% CI, 0.25–0.01; *P*=0.029).

**Conclusions:**

Higher baseline ET-1 concentration was independently associated with worse clinical outcomes and more rapid decline in kidney function. The benefit of dapagliflozin was consistent across the range of ET-1 concentrations measured, and treatment with dapagliflozin led to a small decrease in serum ET-1 concentration.

**Registration:**

URL: https://www.clinicaltrials.gov; Unique identifier: NCT03036124.

**Clinical Perspective**

**What Is New?**

- ET-1 (endothelin-1) has recently been implicated in kidney disease, as it is in heart failure.
- The relationship between ET-1 and renal function in heart failure with reduced ejection fraction has not been described.
- SGLT-2 (sodium-glucose cotransporter-2) inhibitors may inhibit ET-1 secretion in the proximal tubule, raising the possibility of an interaction between ET-1 and these agents.
- In the DAPA-HF trial (Dapagliflozin and Prevention of Adverse Outcomes in Heart Failure), patients with higher baseline ET-1 had higher risk of worsening heart failure and cardiovascular death and significantly steeper decline in kidney function.
- The effect of dapagliflozin was not modified by baseline ET-1 level, and there was a modest reduction in ET-1 level at 12 months with dapagliflozin.

**What Are the Clinical Implications?**

- ET-1 adds prognostic information over and above clinical variables, in addition to NT-proBNP (N-terminal pro-B-type natriuretic peptide) and hs-TnT (high-sensitivity troponin-T), and is associated with the decline in kidney function as well as the risk of hospitalization and death.
- Although there was no interaction between baseline circulating ET-1 and the effect of dapagliflozin, an effect at the proximal tubular level cannot be excluded and may contribute to the renal benefits of SGLT-2 inhibition.
- The reduction in ET-1 with dapagliflozin suggests a new mechanism of action of SGLT-2 inhibition.

The endothelins are a family of 21 amino acid vasoactive peptides consisting of 3 isoforms (ET [endothelin]-1, ET-2, and ET-3) encoded by separate genes.^1–4^ ET-1 is the most abundant and best-characterized isoform.^1–4^ ET-1 is produced in small amounts mainly in endothelial cells in blood vessels and primarily acts as a local paracrine and autocrine mediator. However, under pathophysiological conditions, increased ET-1 production is stimulated in other cell types, including vascular smooth muscle cells, cardiac myocytes, and inflammatory cells.^1^ The effects of ET-1 are mediated by ETA and ETB receptors, which usually have opposing actions. ETA receptors function to promote vasoconstriction and inflammation, whereas ETB receptors produce vasodilation and natriuresis and inhibit inflammation. ET-1 may have diuretic and natriuretic effects in the kidney, mediated predominantly by ETB receptors, leading to inhibition of sodium and chloride reabsorption, suppression of Na^+^/K^+^ ATPase activity, and inhibition of vasopressin-induced water reabsorption in the collecting duct.^2–6^ Recently, a possible role for the endothelins in the progression of kidney dysfunction was suggested by the beneficial effect of the selective ETA receptor antagonist atrasentan in patients with diabetic nephropathy.^7^ These actions are plausibly relevant in heart failure (HF) given the strong bidirectional links between chronic kidney disease and HF. Indeed, circulating ET-1 levels are often elevated in patients with this condition.^8–10^ Moreover, the circulating level of ET-1 is associated with the severity of HF and, in some studies, the risk of HF hospitalization and mortality.^11–14^ However, the relationship between ET-1 levels and serial changes in kidney function in HF has not been reported.

SGLT-2 (sodium-glucose cotransporter-2) is also expressed in the proximal renal tubule, and SGLT-2 inhibitors have demonstrated important cardiovascular and kidney benefits in multiple recent clinical trials,^15–19^ including slowing the rate of decline in estimated glomerular filtration rate (eGFR) in patients with HF.^20^ Intriguingly, the SGLT-2 inhibitor empagliflozin has recently been shown to inhibit basal and IL-1β (interleukin-1β)–induced ET-1 expression in 2 independent human proximal tubular cell lines under normoglycemic conditions, raising the potential for an interaction between the endothelin system and SGLT-2 inhibitors in patients with HF.^21^

We examined the role of serum ET-1 concentration as a prognostic biomarker in a contemporary population with HF and reduced ejection fraction (HFrEF), including its value when added to other established biomarkers, evaluated the relationship between serum ET-1 and decline in kidney function in HFrEF, and investigated whether ET-1 modifies the response to SGLT-2 inhibition in the DAPA-HF trial (Dapagliflozin and Prevention of Adverse Outcomes in Heart Failure).^15^

## Methods

DAPA-HF was a prospective, randomized, double-blind, controlled trial that evaluated the efficacy and safety of 10 mg of dapagliflozin once daily, compared with placebo, added to standard care in 4744 patients with HFrEF followed up for a median of 18.2 months.^15^ Ethics committees at each participating institution approved the protocol, and all patients gave written informed consent. Participation in a prospective biomarker substudy was offered to all enrolled patients in countries where regulations allowed it. The first authors had full access to the data in the study and take responsibility for its integrity and the data analysis. The data that support the findings of this study are available from the corresponding author upon reasonable request.

### Study Patients

Patients ≥18 years of age were eligible if they were in New York Heart Association functional class II to IV, had a left ventricular ejection fraction ≤40%, and were optimally treated with pharmacological and device therapy for HFrEF.^15^ Study participants were also required to have an elevated NT-proBNP (N-terminal pro-B-type natriuretic peptide) level (ie, NT-proBNP ≥600 pg/mL or ≥400 pg/mL if hospitalized for HF within the previous 12 months or ≥900 pg/mL if there was concomitant atrial fibrillation or flutter, irrespective of history of HF hospitalization).

The main exclusion criteria included type 1 diabetes, symptomatic hypotension or systolic blood pressure <95 mm Hg and eGFR <30 mL/min per 1.73 m^2^ or rapid decline in renal function. Patients were also excluded if they had current acute decompensated HF or HF hospitalization within 4 weeks before enrollment, or recent myocardial infarction or coronary revascularization in the preceding 12 weeks.

### Measurement of Serum ET-1 and Other Biomarkers

Venous blood samples were taken at randomization and at 12 months. ET-1 samples were collected in serum tubes, whereas other biomarkers were collected in EDTA anticoagulant tubes. Isolated serum (ET-1) and plasma (other biomarkers) were stored at −20°C or colder until shipped on dry ice to the central repository, where they were stored at −80°C or colder until assayed. ET-1 was measured (TIMI Clinical Trials Laboratory, Boston, MA) using a microfluidics immunoassay on the Ella system (ProteinSimple). The limit of quantitation of the assay is 0.25 pg/mL, with a normal range of 0.92 to 1.58 pg/mL. There were only 5 ET-1 values lower than the limit of quantitation, so no imputation was made. hs-TnT (high-sensitivity troponin-T) was measured (TIMI Clinical Trials Laboratory, Boston, MA) at baseline and 12 months with an Elecsys immunoassay on the Cobas E601 analyzer (Roche Diagnostics).^22^ The limit of quantitation of the assay is 6 ng/L, and the 99th percentile upper reference limit used in the laboratory is 14 ng/L. For analyses as a continuous variable, patients with hs-TnT concentrations <6 ng/L were assigned a value of half the limit of quantitation (ie, 3 ng/L). NT-proBNP was measured at baseline and at 8 months in a central laboratory (Covance) using an Elecsys immunoassay (Roche Diagnostics).^23^

### Prespecified Trial Outcomes

The primary outcome of DAPA-HF was the composite of worsening HF (HF hospitalization or urgent visit for HF) or cardiovascular death, whichever occurred first. Prespecified secondary end points included: HF hospitalization or cardiovascular death; HF hospitalizations (first and recurrent) and cardiovascular deaths; all-cause death; and a change in KCCQ-TSS (Kansas City Cardiomyopathy Questionnaire-total symptom score) from baseline to 8 months. For the KCCQ-TSS, higher scores reflect better health status, and the proportion having a ≥5-point increase or decrease in score at 8 months was determined as previously described.^15^ There was also a prespecified secondary renal composite outcome, but this was not evaluated further in this analysis because of the small number of events.

In addition to these prespecified outcomes, the post hoc outcome of the slope of change in eGFR over time according to baseline ET-1 tertile was calculated as described in the Statistical Analysis section.

### Statistical Analysis

Baseline characteristics were summarized according to baseline ET-1 tertile as mean (SD) or median (interquartile range) for continuous variables and count (percentage) for categorical variables. Differences in the baseline characteristics between tertiles were evaluated with a Wilcoxon-type test for trend.^24^

We analyzed the association between baseline ET-1 tertile and key clinical outcomes, the relationship between change in ET-1 from baseline to 12 months with the primary outcome, the association of ET-1 concentration with changes in renal function, and the efficacy of dapagliflozin according to baseline ET-1 concentrations. In addition, we further investigated the risk of primary and key secondary outcomes according to ET-1 groups by studying the inflexion points in restricted cubic splines. This resulted in 3 ET-1 groups: group 1 (≤4 pg/mL; n=1724), group 2 (>4–7 pg/mL; n=1145), and group 3 (>7 pg/mL; n=179).

Time-to-event end points were analyzed using Kaplan-Meier estimate and Cox proportional-hazards models, with ET-1 modeled as both a categorical variable (tertiles and groups) and continuous variable and stratified according to diabetes status, history of HF hospitalization (except for all-cause death), and treatment group assignment, as described in the trial statistical analysis plan. We further adjusted these estimates using Cox models with known predictors of risk in patients with HF, including age, sex, race, geographic region, duration of HF, heart rate, systolic blood pressure, body mass index, New York Heart Association functional classification, left ventricular ejection fraction, eGFR, etiology of HF, history of atrial fibrillation and NT-proBNP, a model with adjustment for baseline hs-TnT as an additional covariate, as well as an additional model adjusting for baseline use of an angiotensin-converting enzyme inhibitor, angiotensin receptor blocker, angiotensin receptor–neprilysin inhibitor, or mineralocorticoid receptor antagonist. Proportionality of hazards for these models was confirmed visually using log(−log) plots and testing Schoenfeld residuals.

The relationships between baseline ET-1 and risks of key clinical end points were displayed using both unadjusted and adjusted restricted cubic splines with 5 knots. In addition, we described the incidence of the primary outcome according to tertiles of baseline ET-1, tertiles of baseline hs-TnT, and tertiles of baseline NT-proBNP. We then plotted the incidence of the primary outcome according to tertiles of baseline ET-1 versus tertiles of baseline hs-TnT and tertiles of baseline ET-1 versus tertiles of baseline NT-proBNP. The association between change in ET-1 from baseline to 12 months and risk of subsequent outcomes was analyzed in a landmark analysis of patients who were alive at 12 months with available ET-1 data. Hazard ratios (HRs) and 95% CIs for the primary outcome according to the log2-transformed ratio of 12 months to baseline ET-1 were modeled using restricted cubic spline analysis adjusted for log-transformed baseline ET-1, randomized treatment, history of HF hospitalization, and stratified by diabetes status. A repeated-measures mixed-effect model was used to examine the slope of change in eGFR over time and the interaction between treatment and visit, as well as the interaction between baseline ET-1 concentration and visit, with a random intercept and slope per patient as previously described.^20^ The effect of the randomized treatment on change in ET-1 from baseline to 12 months was also examined using an ANCOVA model adjusted for baseline value.

The effect of dapagliflozin compared with placebo on each outcome was calculated as HR and 95% CI derived from Cox proportional-hazards models adjusted for a history of hospitalization for HF and treatment assignment and stratified by baseline diabetes status, as prespecified in the statistical analysis plan for the main trial. The effect of baseline ET-1 concentration on the treatment effect of dapagliflozin compared with placebo was assessed by the inclusion of ET-1 tertile*treatment interaction term in the model, and an interaction *P* value was calculated using a likelihood ratio test. The proportion of patients with a clinically significant (≥5 points) improvement or deterioration in KCCQ-TSS at 8 months was analyzed as previously described and presented as an odds ratio for each ET-1 tertile.^15^

All analyses were conducted using Stata version 16.0 (StataCorp, College Station, TX) and SAS version 9.4 (SAS Institute, Cary, NC). A *P* value <0.05 was considered statistically significant.

## Results

Baseline serum ET-1 was measured in 3048 patients, and 12-month serum ET-1 was measured in 2436 patients. The median baseline ET-1 concentration was 3.81 pg/mL (25th to 75th percentile, 3.03–4.80), with tertile 1 ≤3.28 pg/mL (n=1016), tertile 2 >3.28 to 4.41 pg/mL (n=1022), and tertile 3 >4.41 pg/mL (n=1010).

### Baseline Characteristics

Baseline characteristics according to ET-1 tertiles are summarized in Table 1. Patients with higher baseline ET-1 concentrations were more likely to be male, non-Asian, and obese, with more comorbidities, especially diabetes, atrial fibrillation, and chronic obstructive pulmonary disease (Table 1). They also had worse kidney function, lower left ventricular ejection fraction, and poorer functional status, with a higher proportion of patients with New York Heart Association class III or IV symptoms and lower (worse) KCCQ-TSS (each *P*<0.001). Patients with higher baseline ET-1 were more often treated with a diuretic, an angiotensin receptor–neprilysin inhibitor, digoxin, and devices but less often with a mineralocorticoid receptor antagonist.

**Table 1.**
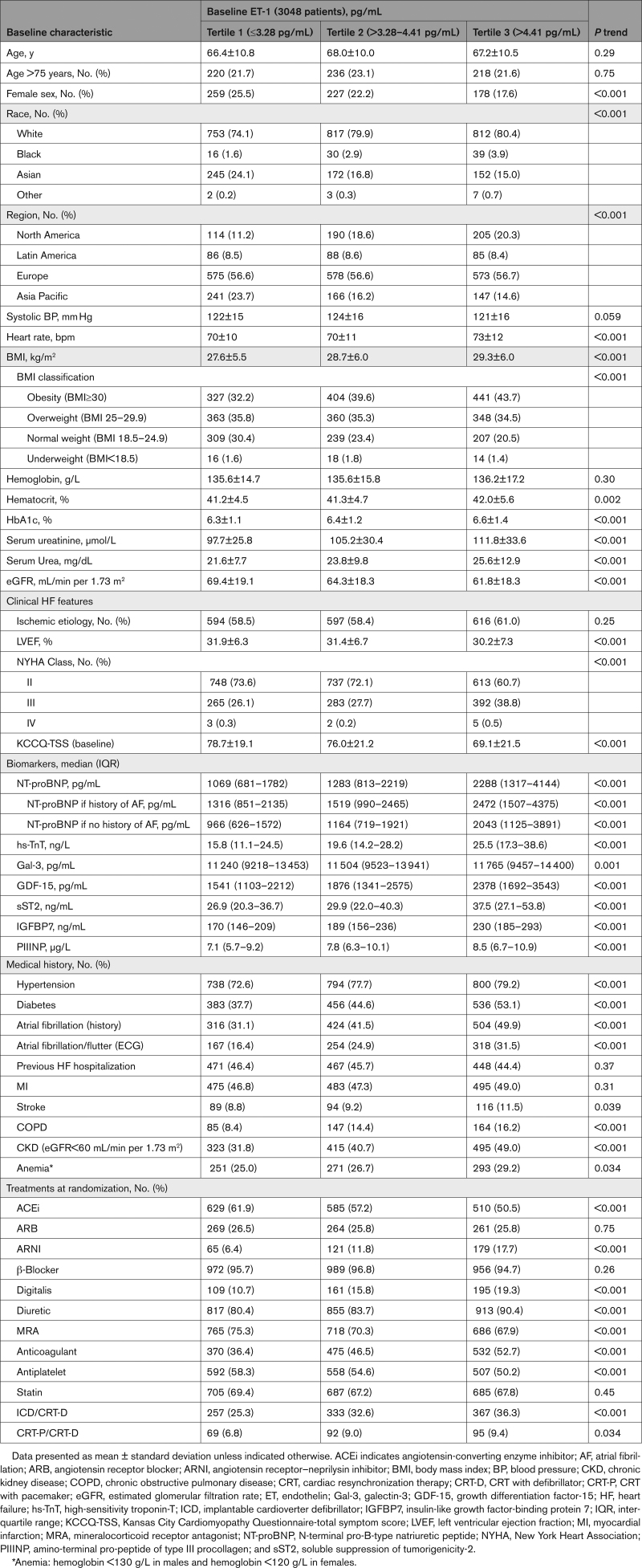
Patient Characteristics According to Baseline ET-1 Tertile

### Relationship Between ET-1 and Clinical Outcomes

**Figure 1.**
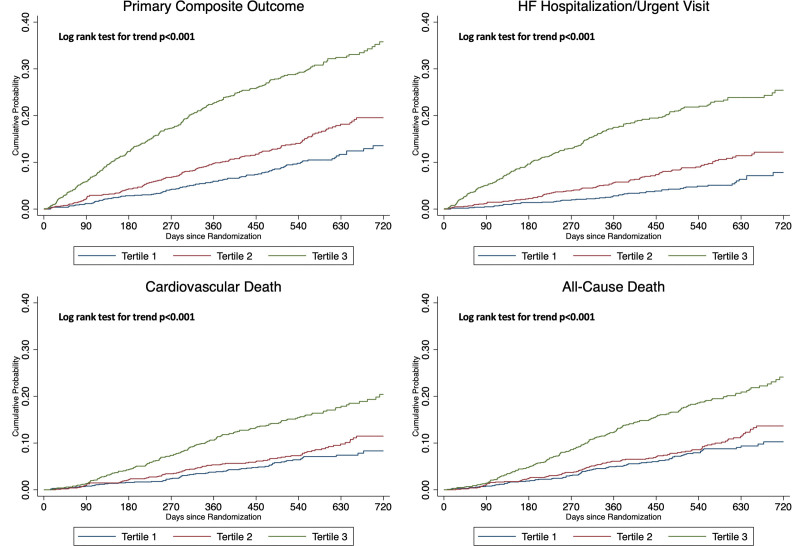
**Kaplan-Meier curves showing key trial outcomes according to baseline ET-1 (endothelin-1) tertile.** The primary outcome was a composite of worsening heart failure (hospitalization or an urgent visit resulting in intravenous therapy for heart failure) or death from cardiovascular causes.

Incidence rates of the primary and secondary outcomes increased with increasing ET-1 tertile, with the difference most marked for the outcome of worsening HF (Table 2; Figure 1). The elevated risk for the primary end point remained significant after comprehensive adjustment for prognostic variables, including NT-proBNP, with an adjusted HR (aHR) of 1.36 (95% CI, 1.06–1.75) for tertile 2 and 1.95 (95% CI, 1.53–2.50) for tertile 3, compared with tertile 1. This higher risk was driven by the risk of worsening HF, with an aHR of 1.54 (95% CI, 1.10–2.18) and 2.54 (95% CI, 1.82–3.53) for tertiles 2 and 3, respectively. However, the risk relationship with death was attenuated by adjustment, and the aHR for all-cause mortality was 1.08 (95% CI, 0.80–1.45) and 1.45 (95% CI, 1.09–1.93) for tertiles 2 and 3, respectively. This pattern of risk was maintained after additional adjustment for baseline hs-TnT (Table 2).

**Table 2.**
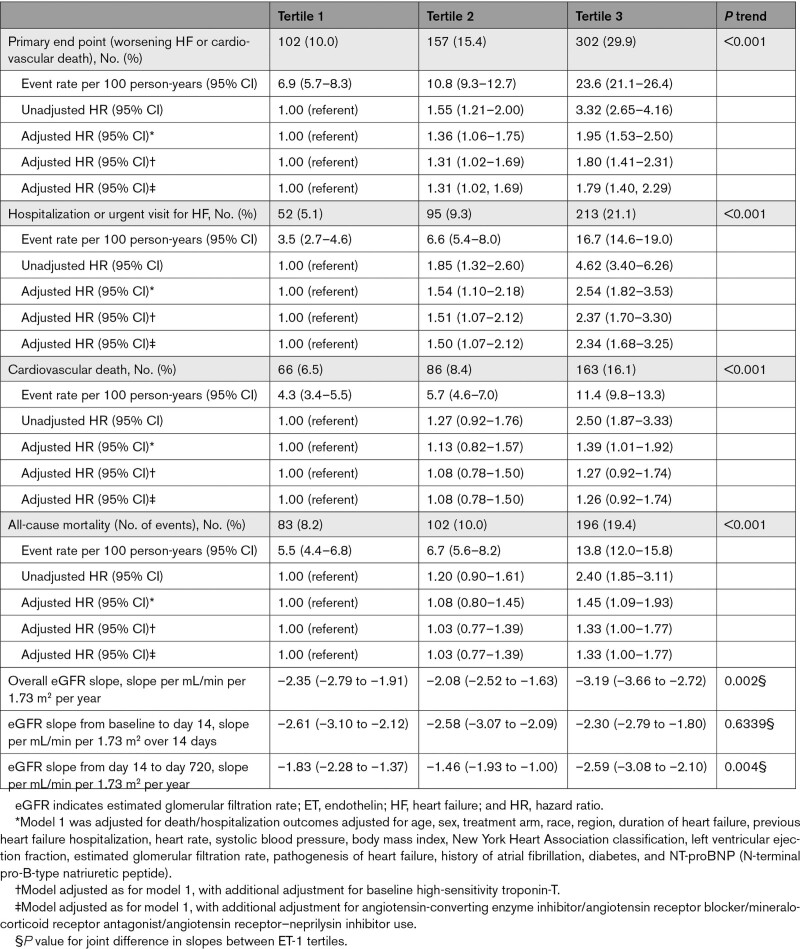
Event Rate (per 100 Person-Years) and Hazard Ratios for Trial Outcomes According to Baseline ET-1 Tertile

**Figure 2.**
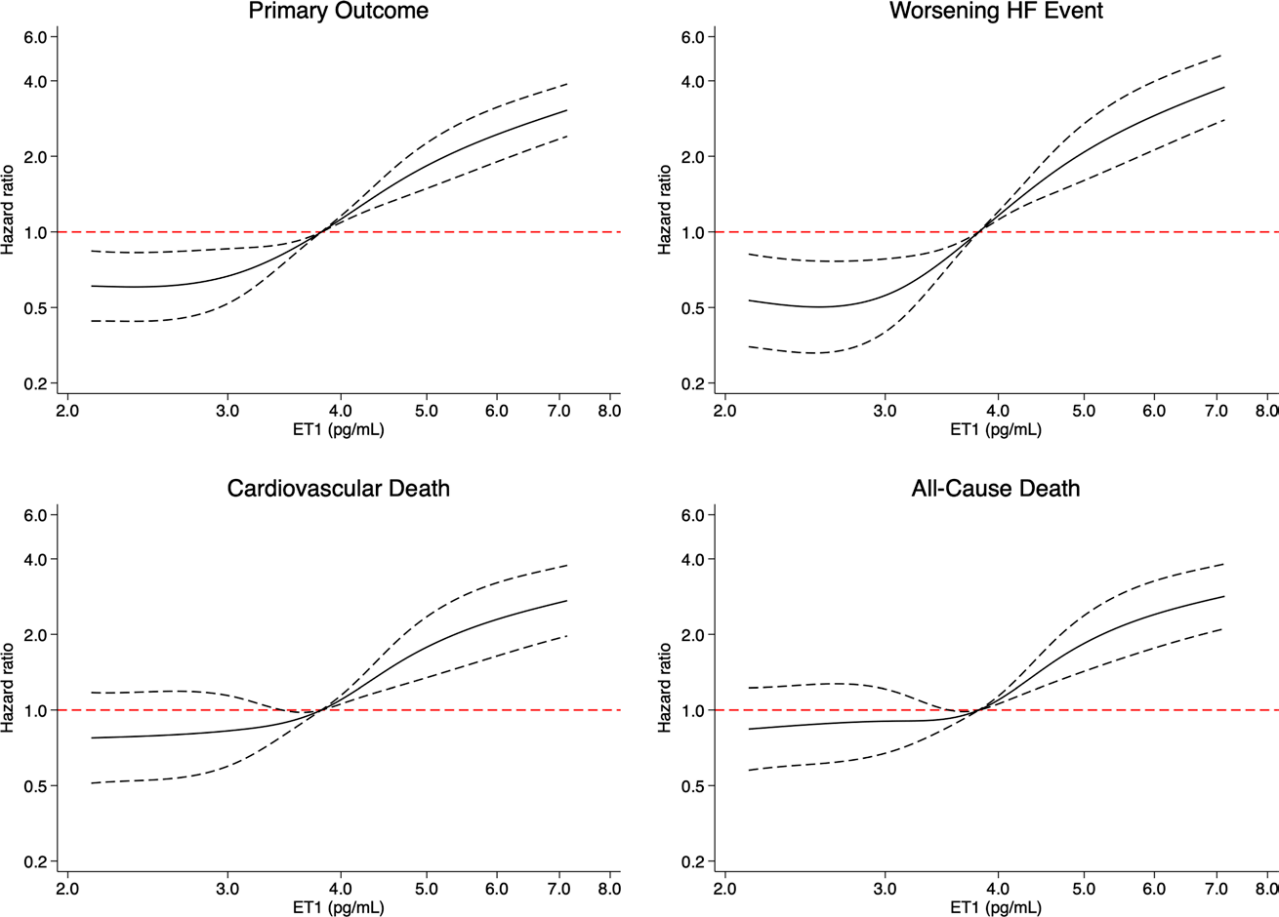
**Key trial outcomes according to baseline ET-1 levels.** These restricted cubic splines demonstrate the unadjusted risk of each outcome modeling baseline log-transformed ET-1 levels as a continuous variable. The interrupted lines represent corresponding 95% CIs. ET-1 indicates endothelin-1; and HF, heart failure.

Inspection of the restricted cubic spline models suggested a linear increase in the risk of the primary and secondary outcomes from an ET-1 concentration >4 pg/mL which, when log-transformed, equates to log ET-1 >1.39 pg/mL (Figure 2; Figure S1). Analysis of the primary and secondary outcomes according to the ET-1 group (group 1: 0–4 versus group 2: >4–7 versus group 3: >7 pg/mL) showed a more graduated increase in risk compared with an analysis by tertiles (Table S1; Figure S2) with an HR for the primary outcome, adjusted for predictive variables including baseline NT-proBNP and hs-TnT, of 1.51 (95% CI, 1.24–1.83) for group 2 and 2.28 (95% CI, 1.68–3.11) for group 3 compared with group 1, and an aHR for all-cause mortality of 1.28 (95% CI, 1.01–1.62) and 1.57 (95% CI, 1.08–2.28) for groups 2 and 3, respectively.

**Figure 3.**
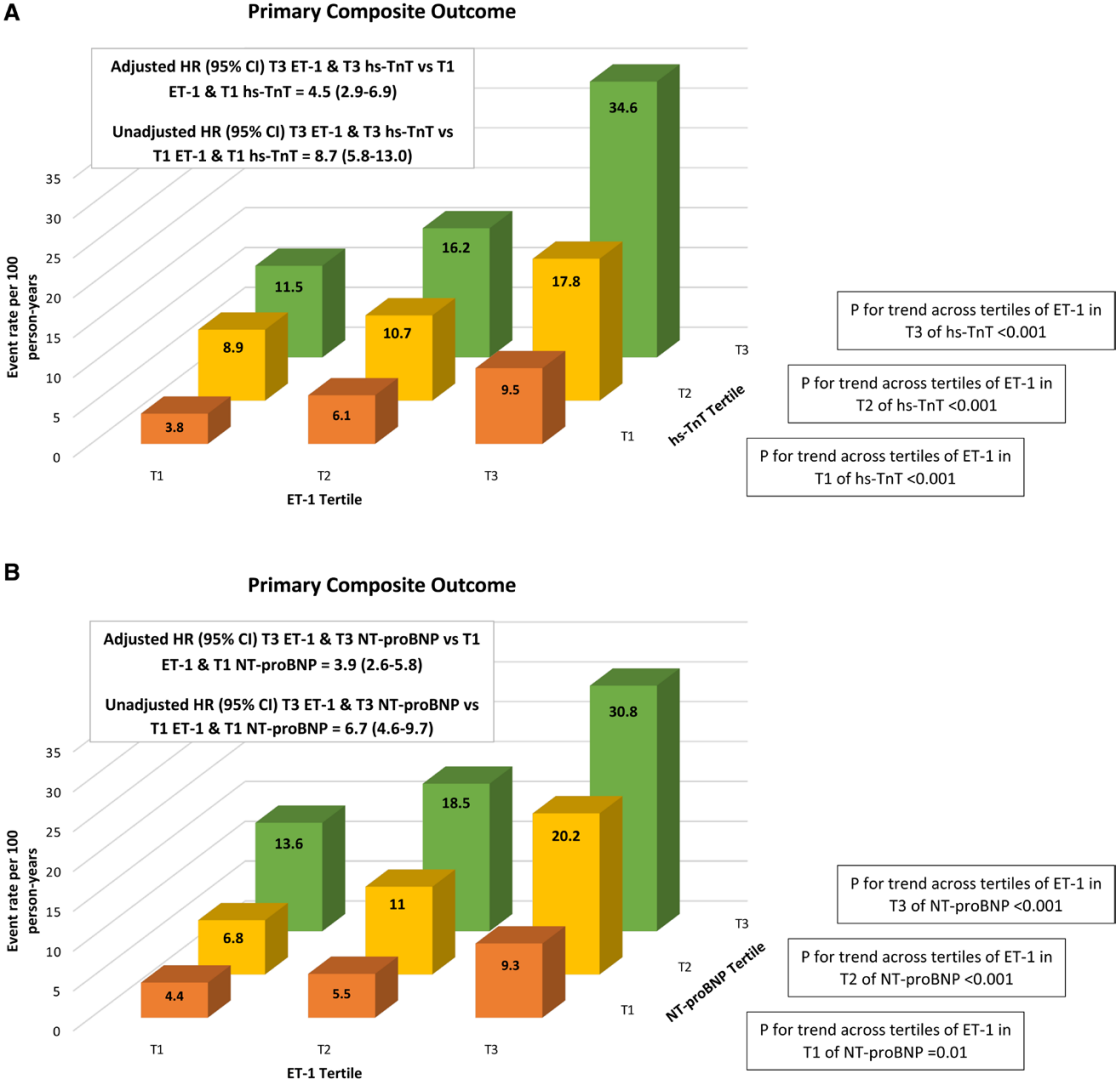
**Event rates for the primary outcome according to baseline ET-1, hs-TnT, and NTproBNP.** A, Baseline ET-1 and hs-TNT tertiles. B, Baseline ET-1 and NTproBNP tertiles. ET-1 indicates endothelin-1; HR, hazard ratio; hs-TnT, high-sensitivity troponin-T; NT-proBNP, N-terminal pro-B-type natriuretic peptide; and T, tertile.

The additive risk of ET-1 and hs-TnT is illustrated in Figure 3A, which shows a >8-fold higher risk for patients in tertile 3 for both ET-1 and hs-TnT compared with those in tertile 1 for both peptides (event rate per 100 person-years, 34.6 versus 3.8). A similar pattern was seen when baseline ET-1 was analyzed together with baseline NT-proBNP, with a >6-fold higher risk for patients in tertiles 3 of both ET-1 and NT-proBNP compared with patients in tertile 1 for both biomarkers (event rate per 100 person-years, 30.8 versus 4.4; Figure 3B).

### Relationship Between Baseline ET-1 and Change in Kidney Function

**Figure 4.**
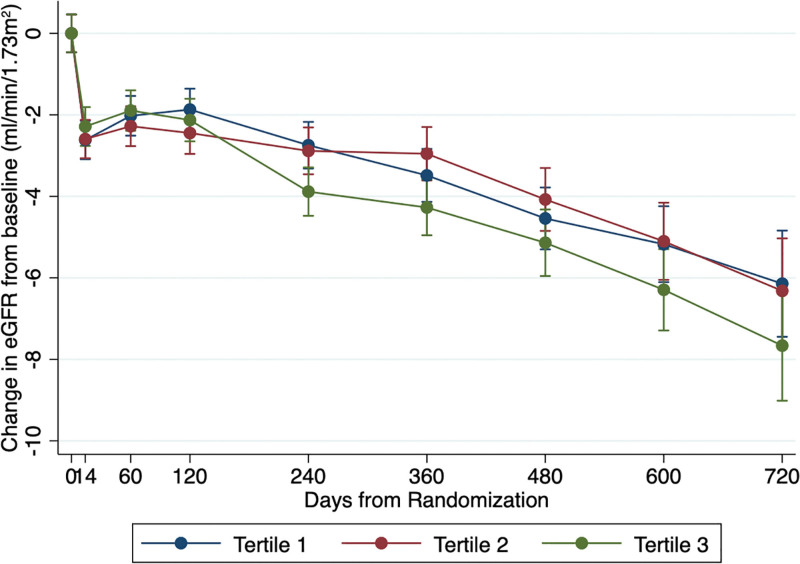
**Change in eGFR from baseline in DAPA-HF according to baseline ET-1 (endothelin-1) tertile.** The change in eGFR from baseline at each time point is displayed as mean with 95% CI. DAPA-HF indicates Dapagliflozin and Prevention of Adverse Outcomes in Heart Failure trial; and eGFR, estimated glomerular filtration rate.

Overall, kidney function declined during follow-up in DAPA-HF. The steepest rate of decline in eGFR was in ET-1 tertile 3. The overall eGFR slope, measured as mL/min per 1.73 m^2^ per year, was –2.35 (95% CI, –2.79 to –1.91) in tertile 1, –2.08 (95% CI, –2.52 to –1.63) in tertile 2, and –3.19 (95% CI, –3.66 to –2.72) in tertile 3 (*P*=0.002; Table 2, Figure 4).

### Effect of Dapagliflozin on Primary and Secondary Trial Outcomes According to Baseline ET-1 Concentration

Of the 3048 patients with baseline ET-1 measurements, dapagliflozin reduced the primary outcome of cardiovascular death or worsening HF by 22% (HR, 0.78 [95% CI, 0.66–0.92]). The efficacy of dapagliflozin in preventing the primary end point was consistent regardless of baseline ET-1 concentration, whether analyzed according to tertiles (*P*-interaction=0.47) or as a continuous variable (*P*-interaction=0.10; Table 3; Figure S3). Similarly, there was no difference in the treatment effect of dapagliflozin on preventing HF hospitalizations or urgent HF visits, cardiovascular death, and all-cause deaths according to baseline ET-1 tertiles.

**Table 3.**
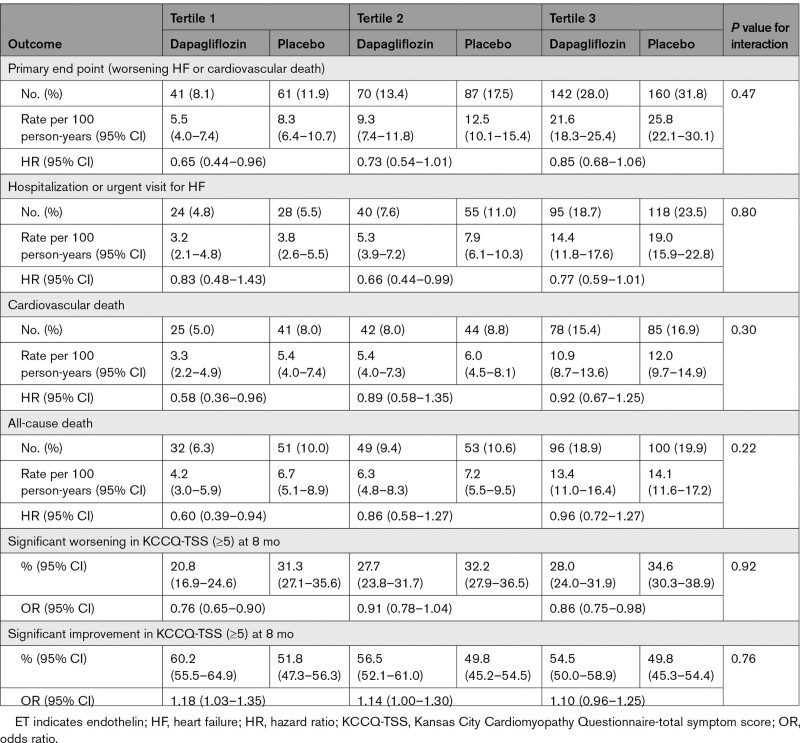
Effect of Dapagliflozin on the Primary and Secondary Outcomes According to Baseline ET-1 Tertile

### Effect of Dapagliflozin on eGFR Slope According to Baseline ET-1 Concentration

Compared with placebo, dapagliflozin resulted in an initial decrease in eGFR overall, from baseline to day 14, and this was similar across all ET-1 tertiles. Thereafter, the rate of decline in eGFR was steeper in the placebo group than in the dapagliflozin group overall. This pattern was also the case across each tertile of ET-1, with the fastest rate of decline in the third of patients with the highest ET-1 level at baseline (Figure S4). For ET tertile 1, the change in eGFR in the dapagliflozin group between day 14 and day 720 was –1.11 (95% CI, –1.72 to –0.50) mL/min per 1.73 m^2^ per year compared with –2.53 (95% CI, –3.14 to –1.92) in the placebo group (*P* for difference=0.0013). For tertile 2, change in eGFR was –0.55 (95% CI, –1.14 to 0.05) in the dapagliflozin group versus –2.43 (95% CI, –3.05 to –1.82) in the placebo group (*P* for difference<0.001). For tertile 3, change in eGFR was –1.75 (95% CI, –2.52 to –0.97) in the dapagliflozin group versus –3.62 (95% CI, –4.41 to –2.83) in the placebo groups (*P* for difference=0.0009). The interaction *P* value between ET-1 tertile and treatment group was 0.12.

### Change in ET-1 Concentration Between Baseline and 12 Months

Compared with placebo, there was a reduction in ET-1 level at 12 months with dapagliflozin (difference –0.13 pg/mL [95% CI, –0.25 to -0.01]; *P*=0.029]. In the placebo group, ET-1 level increased (0.10 pg/mL [95% CI, 0.003–0.20]) from baseline to 12 months, whereas in the dapagliflozin group, ET-1 decreased (–0.04 pg/mL [95% CI, –0.13 to 0.05]).

Figure S5 displays the association between change in ET-1 assessed as a continuous variable and subsequent outcomes: a doubling of ET-1 from baseline to 12 months was associated with an HR, for the primary composite of 3.23 (95% CI, 2.18–4.79). Conversely, a halving of ET-1 from baseline was associated with an HR of 0.38 (95% CI, 0.21–0.66).

### Safety and Adverse Events

Inspection of the placebo group suggested that the rate of discontinuation of randomized treatment increased as ET-1 increased, as did the frequency of renal adverse events. No other adverse event appeared to show any association with ET-1 level at baseline. Generally, there was no clear difference between placebo and dapagliflozin for any adverse event according to ET-1 tertile (Table S2).

## Discussion

We believe that this is the largest-ever study of the association between circulating ET-1 and a range of outcomes in HFrEF.^25^ We confirmed the prognostic importance of this peptide in a well-treated, contemporary population, provided novel information about the incremental predictive value of ET-1 when added to NT-proBNP and hs-TnT (particularly for HF hospitalization), and showed a previously unknown association between ET-1 and progressive worsening of kidney function over time in patients with HFrEF. We also showed that despite a potential interaction between ET-1 and SGLT-2 in the proximal renal tubule, the benefits of dapagliflozin were consistent across the range of serum ET-1 concentrations measured in DAPA-HF.

Although discovered in 1988, less is known about the clinical importance of ET-1 than most other neurohumoral biomarkers in HF.^26^ By far, the largest previous report about this peptide in patients with chronic HFrEF was from the neurohumoral substudy of the Val-HeFT (Valsartan Heart Failure Trial), which included 1934 participants enrolled in the United States.^27^ Although details of baseline treatment were not provided in this report, in the parent trial, conducted between 1997 and 2000, just more than one-third of patients were treated with a beta-blocker, and ≈4% were treated with a mineralocorticoid receptor antagonist.^28^ Although this and some other studies included natriuretic peptides, none reported measurement of ET-1 and troponin, which has emerged as another incrementally important prognostic marker in contemporary trials.^22,29^ In Val-HeFT, a baseline ET-1 level ≥1.50 pmol/L was a univariate predictor of morbidity and mortality, although its prognostic value in a multivariable model was not reported. In our larger study of patients receiving contemporary therapy, serum ET-1 concentration was associated with the primary composite outcome, its components, and all-cause mortality, both in univariate and multivariate analyses. Importantly, ET-1 remained independently associated with these outcomes, even in models including NT-proBNP and the combination of NT-proBNP and hs-TnT in addition to other prognostic clinical variables, an attribute shared by few if any other biomarkers.^30,31^ Speculatively, ET-1 might be associated with worse outcomes given that it is a much more potent vasoconstrictor than angiotensin II, on a molar basis, and a powerful mitogen known to cause hypertrophy and fibrosis.^1–4,8,9^ The vasoconstrictor actions of ET-1, mediated by the ETA receptor, may be most pronounced in the pulmonary circulation, and endothelin receptor antagonists have been developed as an important treatment for patients with primary pulmonary hypertension.^1–4^ These ET-1 mechanisms may in part explain the strong association we observed with HF hospitalization, through the worsening of symptoms. Unfortunately, endothelin receptor antagonists have not been effective in patients with HFrEF. Indeed, in virtually all placebo-controlled trials, endothelin receptor antagonists caused worsening HF symptoms and signs.^32–34^ This unexpected outcome has never been adequately explained. However, these agents cause fluid retention with the postulated mechanism being cross-talk between ETA and ETB receptors, resulting in a degree of ETB receptor blockade even with specific ETA blockers, and this adverse effect may be dose related and differ by receptor antagonist selectivity.^35,36^

Although endothelin receptor antagonists were not beneficial in HF, the selective ETA antagonist atrasentan has recently been shown to slow the rate of decline in kidney function in patients with diabetes and chronic kidney disease.^7^ Consequently, we also examined the relationship between serum ET-1 concentration and the rate of decline in eGFR over time among patients included in DAPA-HF. Patients in the highest ET-1 tertile in DAPA-HF had a significantly greater rate of decrease in eGFR compared with patients in tertile 1, suggesting that ET-1 might also play a role in the progressive decline in kidney function that occurs in many patients with HF.^37^ Ongoing clinical trials in patients with chronic kidney disease will define the future position of endothelin receptor antagonists in the management of chronic kidney disease.^38,39^

Because of the known potent vasoconstrictor properties of ET-1, the focus of the potential actions of this peptide in the kidney has been on renal blood flow and glomerular hemodynamics. However, as alluded to above, ET-1 also plays a role in sodium and water homeostasis, and ET-1 levels correlate with markers of congestion in patients with HF.^5,40,41^ These findings, along with the fact that ET-1 acts in the proximal renal tubule and experimental evidence that SGLT-2 inhibition reduces ET-1 expression in human proximal tubular cell lines, raised the possibility of an interaction between ET-1 level and the effects of dapagliflozin in patients with HFrEF.^21^ However, we did not find evidence for this in DAPA-HF. The benefit of dapagliflozin was consistent across the range of ET-1 concentrations measured. Nevertheless, there is interest in the combination of an SGLT-2 inhibitor (causing diuresis and a rise in hematocrit) and endothelin receptor antagonists (causing fluid retention and a decrease in hematocrit) because of their complementary actions.^42^

The modest reduction in ET-1 levels at 12 months with dapagliflozin was notable, although the explanation for this effect is unknown. It might be indirect, with a secondary reflex reduction caused by overall improvement in HF status, or reflect a direct action of SGLT-2 inhibition on the secretion of ET-1 from the blood vessel wall or elsewhere. This finding raises the possibility that some of the renal and even other benefits of SGLT-2 inhibition might be a result of a reduction in ET-1.

### Limitations

This was not a prespecified analysis of the DAPA-HF trial. Because of the inclusion and exclusion criteria, these findings cannot be generalized to patients with mildly reduced and preserved ejection fraction and patients with severely reduced eGFR. We had only one follow-up measurement of ET-1 12 months after randomization, meaning that we could not look at short-term changes in ET-1, and the 12-month measurement was, by definition, in a survivor cohort. The interpretation of systemic circulating ET-1 levels is difficult because ET-1 is a locally secreted and acting peptide, and blood levels reflect “spill-over” from tissues. Measurement of big ET-1 as well as ET-1 would have provided additional pathophysiological insights including secretion of the precursor peptide and endothelin-converting enzyme activity.

### Conclusions

Elevated serum ET-1 concentration was associated with worse clinical outcomes in a contemporary, well-treated cohort of patients with HFrEF, independently of other prognostic variables including NT-proBNP and hs-TnT. Baseline ET-1 concentration was also associated with a more rapid decline in kidney function. The benefit of dapagliflozin was consistent across the range of ET-1 concentrations measured, and treatment with dapagliflozin led to a small reduction in ET-1.

## Article Information

### Sources of Funding

The DAPA-HF trial was funded by AstraZeneca. J.J.V.M. and P.S.J. are supported by a British Heart Foundation Centre of Research Excellence grant RE/18/6/34217 and the Vera Melrose Heart Failure Research Fund.

### Disclosures

K.F.D. reported that his employer, the University of Glasgow, has been remunerated by AstraZeneca for working on the DAPA-HF and DELIVER trials. He has received speaker honoraria from AstraZeneca and Radcliffe Cardiology, has served on an advisory board for Us2.ai and Bayer AG, served on a clinical end point committee for Bayer AG, and has received research grant support from Boehringer Ingelheim, AstraZeneca, and Novartis (paid to his institution). R.T.C reports speaker honoraria from AstraZeneca and has served on an advisory board for Bayer AG. P.S.J reported his employer being paid by AstraZeneca for his time working on the study and receiving personal fees from, and his employer being paid by, Novartis; grants and personal fees from Boehringer Ingelheim; personal fees from Cytokinetics and Vifor Pharma outside the submitted work; and being the director of Global Clinical Trials Partners Ltd. A.H. is an employee of AstraZeneca. H.J.L.H reports grant funding and honoraria for consultancy as a member of the steering committee of the DAPA-CKD trial paid to his institution from AstraZeneca; research grants paid to his employer from AstraZeneca, Boehringer Ingelheim, Janssen, and Novo Nordisk for clinical trials; consulting fees, paid to his employer from Abbvie, Boehringer Ingelheim, Travere Pharmaceuticals, and Novo Nordisk; fees for steering committee membership paid to his employer from Bayer, Chinook, CSL Pharma, Janssen, and Gilead; honoraria for lectures from AstraZeneca and Mitsubishi Tanabe; and has received honoraria for advisory board participation for Merck (paid to his employer), Mitsubishi Tanabe, and Mundipharma. P.J. reports research support from Abbott Laboratories, Amgen, Inc, AstraZeneca, LP, Daiichi-Sankyo, Inc, GlaxoSmithKline, Merck & Co., Inc, Regeneron, Roche Diagnostics Corporation, and Siemens Healthineers. L.K. has received other support from AstraZeneca and personal fees from Novartis and Bristol Myers Squibb as a speaker. M.N.K. reported receiving grants and personal fees from AstraZeneca and Boehringer Ingelheim and personal fees from Sanofi, Amgen, Novo Nordisk, Merck, Eisai, Janssen, Bayer, GlaxoSmithKline, Glytec, Intarcia, Novartis, Applied Therapeutics, Amarin, and Eli Lilly outside the submitted work. F.A.M. reported receiving personal fees from AstraZeneca during the conduct of the study. P.P. reported receiving personal fees and fees to his institution for participation as an investigator in clinical trials from AstraZeneca during the conduct of the study and from Boehringer Ingelheim, Servier, Novartis, Berlin-Chemie, Bayer, Renal Guard Solutions, Pfizer, Respicardia, Cardiorentis, and Cibiem; grants, personal fees, and fees to his institution from Impulse Dynamics; and fees to his institution from Vifor, Corvia, and Revamp Medical outside the submitted work. S.D.S. reported receiving grants from AstraZeneca during the conduct of the study and grants from Alnylam, Amgen, AstraZeneca, Bellerophon, Bayer, Bristol Myers Squibb, Celladon, Cytokinetics, Eidos, Gilead, GlaxoSmithKline, Ionis, Lone Star Heart, Mesoblast, MyoKardia, National Institutes of Health/National Heart, Lung, and Blood Institute, Novartis, Sanofi Pasteur, and Theracos; and personal fees from Akros, Alnylam, Amgen, Arena, AstraZeneca, Bayer, Bristol Myers Squibb, Cardior, Corvia, Cytokinetics, Daiichi-Sankyo, Gilead, GlaxoSmithKline, Ironwood, Merck, Myokardia, Novartis, Roche, Takeda, Theracos, Quantum Genetics, Cardurion, AoBiome, Janssen, Cardiac Dimensions, and Tenaya outside the submitted work. M.S., O.B., and P.J.G. are employees and/or shareholders of AstraZeneca. N.S. reports personal fees from Afimmune, Amgen, Eli Lilly, Hanmi Pharmaceuticals, Merck Sharp & Dohme, Novo Nordisk, Pfizer, and Sanofi; grants and personal fees from AstraZeneca, Boehringer Ingelheim, and Novartis; and a grant from Roche Diagnostics, outside the submitted work. P.W. reports grant income from Roche Diagnostics, AstraZeneca, Boehringer Ingelheim, and Novartis; and personal fees from Novo Nordisk outside the submitted work. M.S.S. reported receiving grants and personal fees from AstraZeneca during the conduct of the study; grants and personal fees from Amgen, Intarcia, Janssen Research and Development, Medicines Company, MedImmune, Merck, and Novartis; personal fees from Anthos Therapeutics, Bristol Myers Squibb, CVS Caremark, DalCor, Dyrnamix, Esperion, IFM Therapeutics, and Ionis; and grants from Daiichi-Sankyo, Bayer, Pfizer, Poxel, Eisai, GlaxoSmithKline, Quark Pharmaceuticals, and Takeda outside the submitted work; and is a member of the TIMI Study Group, which has also received institutional research grant support through Brigham and Women’s Hospital from Abbott, Aralez, Roche, and Zora Biosciences. D.A.M. reports grants to the TIMI Study Group from Abbott Laboratories, Amgen, AnthosTherapeutics, AstraZeneca, BRAHMS, Eisai, GlaxoSmithKline, Medicines Company, Merck, Novartis, Pfizer, Roche Diagnostics, Quark, Siemens, and Takeda, and consultant fees from InCardia, Merck & Co, Novartis, and Roche Diagnostics. J.J.V.M. reports payments through Glasgow University from work on clinical trials, consulting, and other activities from Amgen, AstraZeneca, Bayer, Cardurion, Cytokinetics, GSK, KBP Biosciences, and Novartis; and personal consultancy fees from Alnylam Pharma, Bayer, BMS, George Clinical PTY Ltd, Ionis Pharma, Novartis, Regeneron Pharma, River 2 Renal Corporation; personal lecture fees from Abbott, Alkem Metabolics, Astra Zeneca, Blue Ocean Scientific Solutions Ltd, Boehringer Ingelheim, Canadian Medical and Surgical Knowledge, Emcure Pharma Ltd, Eris Lifesciences, European Academy of CME, Hikma Pharmaceuticals, Imagica Health, Intas Pharma, J.B. Chemicals & Pharma Ltd, Lupin Pharma, Medscape/Heart.Org, ProAdWise Communications, Radcliffe Cardiology, Sun Pharma, the Corpus, Translation Research Group, and Translational Medicine Academy. He is a director of Global Clinical Trial Partners Ltd. The other authors report no conflicts.

### Supplemental Material

Supplemental Methods

Figures S1–S5

Tables S1 and S2

## Supplementary Material
