# Supplementary material for: Endothelin-1, Outcomes in Patients With Heart Failure and Reduced Ejection Fraction, and Effects of Dapagliflozin: Findings From DAPA-HF
Source: Circulation. 2023 Apr 11;147(22):1670–83. doi: 10.1161/CIRCULATIONAHA.122.063327 (PMC10212584; doi:10.1161/CIRCULATIONAHA.122.063327)
Supplement: Supplementary file 1 [file cir-147-1670-s001.pdf]

SUPPLEMENTAL MATERIAL

SUPPLEMENTAL FIGURES

Supplementary Figure S1: Key trial outcomes according to baseline ET-1 levels.

Caption: These restricted cubic splines demonstrate the adjusted risk of each outcome modeling baseline ET-1 levels as a continuous variable. The interrupted lines represent corresponding 95% confidence intervals.

Supplementary Figure S2: Kaplan-Meier curves showing key study outcomes according to baseline ET-1 group (group 1: 0-4 vs group 2: >4-7 vs group 3: >7 pg/mL)

Supplementary Figure S3: Kaplan-Meier curves showing the effect of treatment group on the primary outcome according to ET-1 tertile

Supplementary Figure S4: Effect of dapagliflozin (vs. placebo) on change in eGFR from baseline, according to baseline ET-1 tertiles

Supplement Figure S5: Association between change in ET-1 from baseline to 12 months and subsequent risk of the primary outcome

Supplementary Figure 1: Key trial outcomes according to baseline ET-1 levels. These restricted cubic splines demonstrate the risk of each outcome modeling baseline ET-1 concentration as a continuous variable, adjusted for prognostic variables. The interrupted lines represent corresponding 95% confidence intervals.

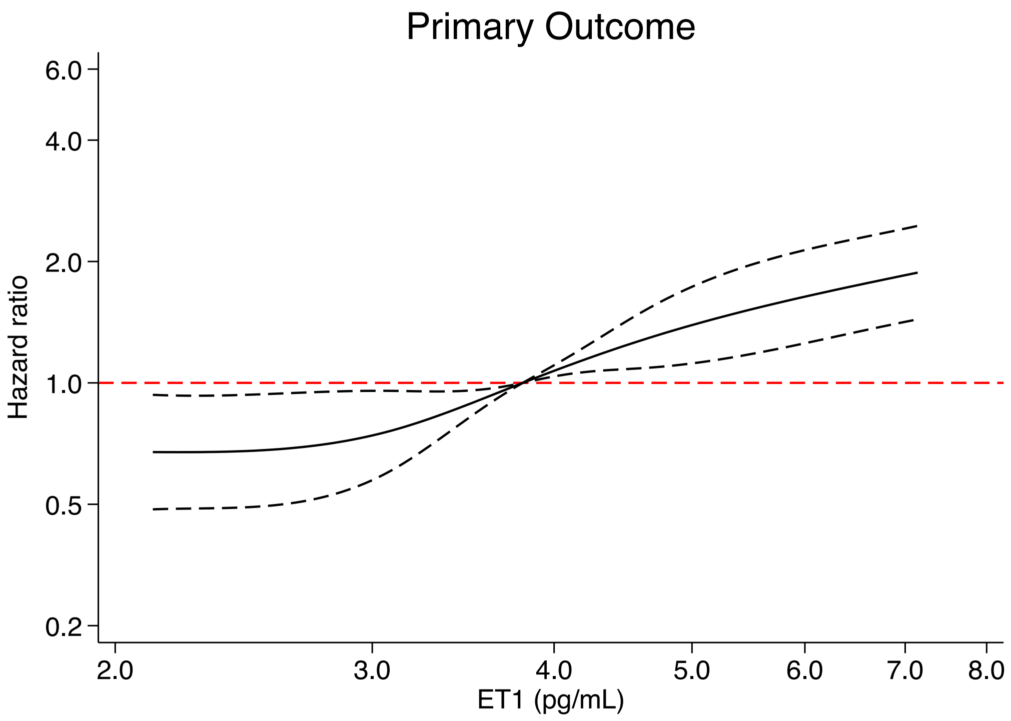

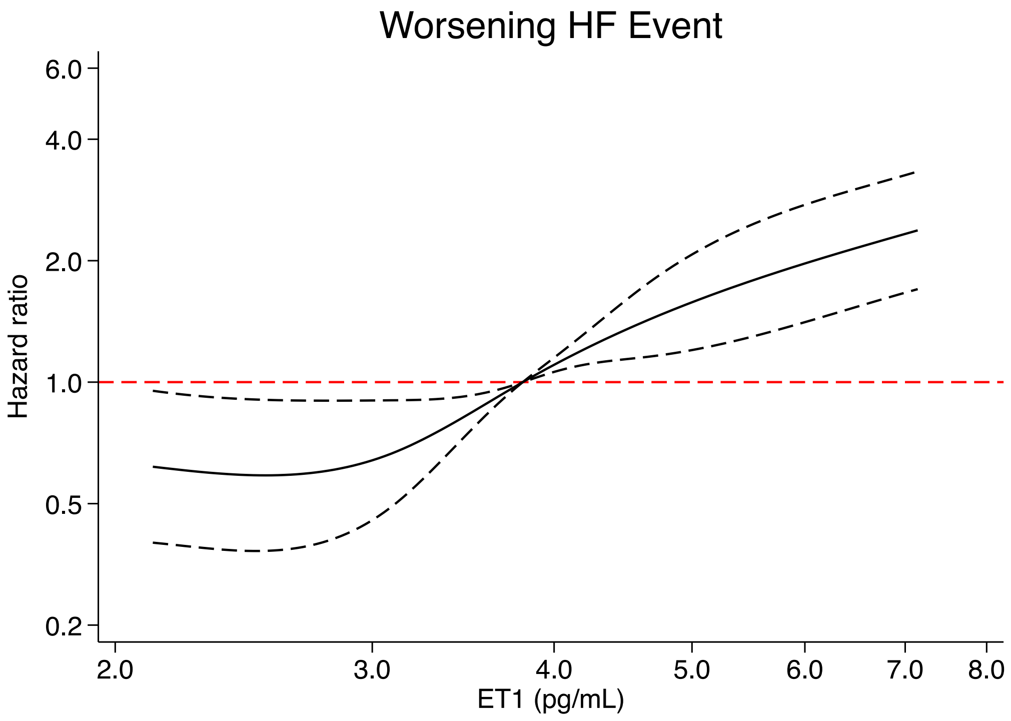

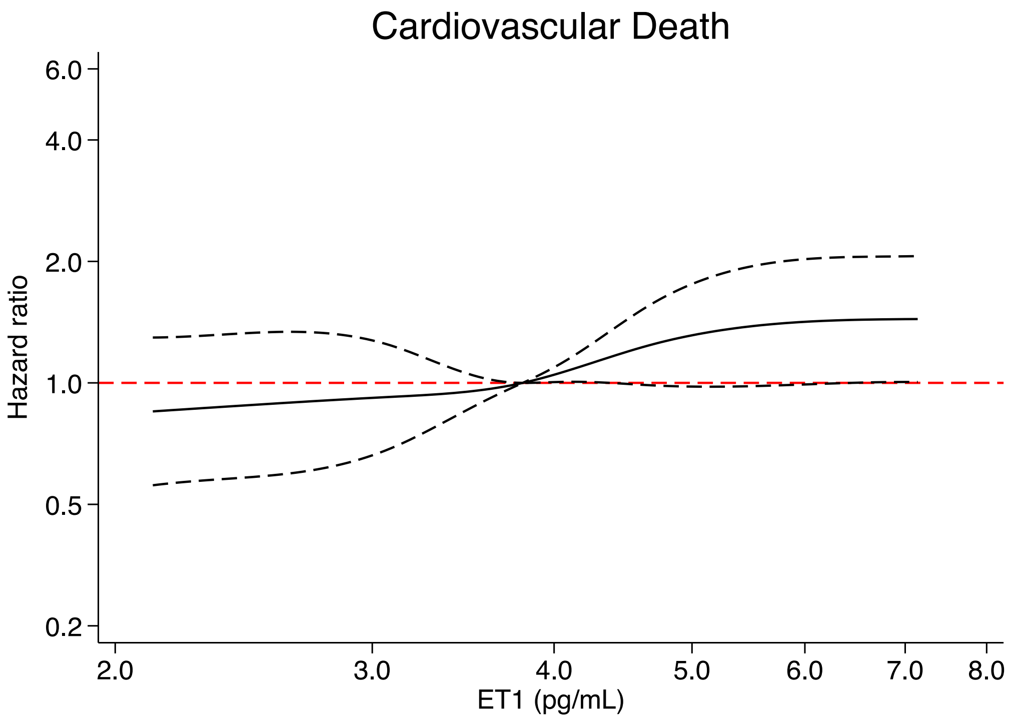
Cardiovascular Death
Hazard ratio
6.0
4.0
2.0
1.0
0.5
0.2
2.0
3.0
4.0
5.0
6.0
7.0
8.0
ET1 (pg/mL)

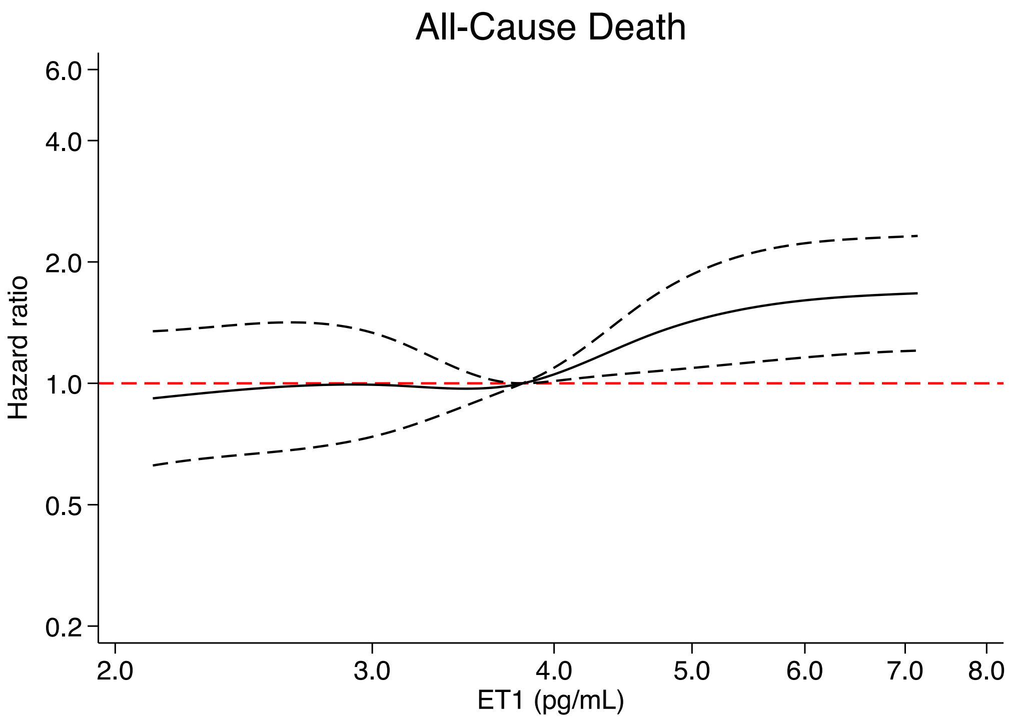
All-Cause Death
Hazard ratio
6.0
4.0
2.0
1.0
0.5
0.2
2.0
3.0
4.0
5.0
6.0
7.0
8.0
ET1 (pg/mL)

Supplementary Figure 2: Kaplan-Meier curves showing key study outcomes according to baseline ET-1 group (group 1: 0-4 vs group 2: >4-7 vs group 3: >7 pg/mL)

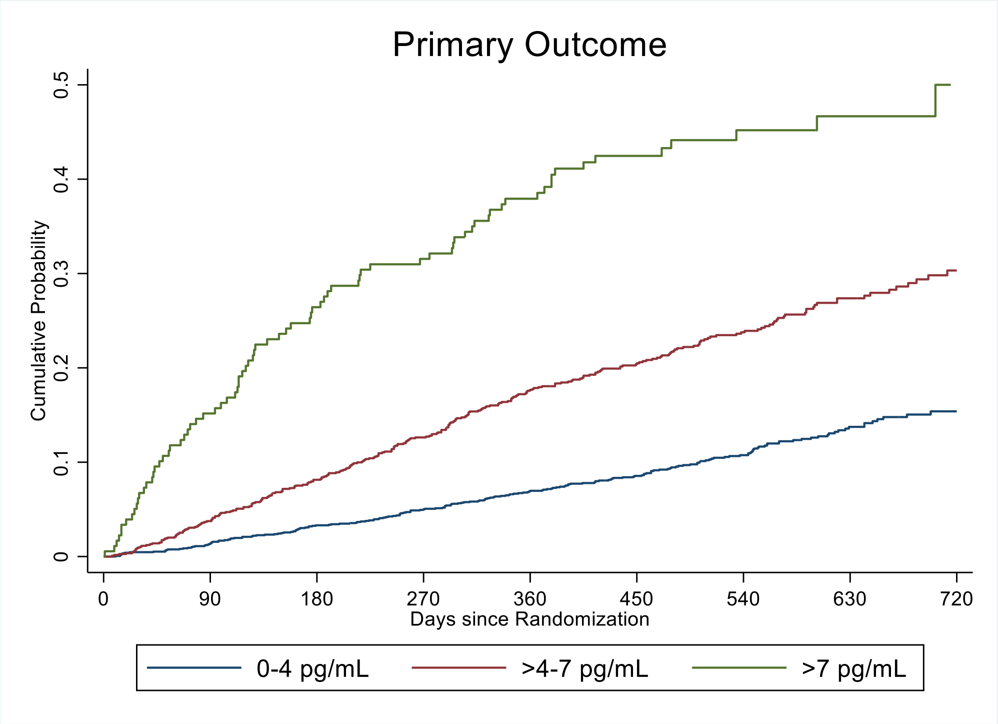

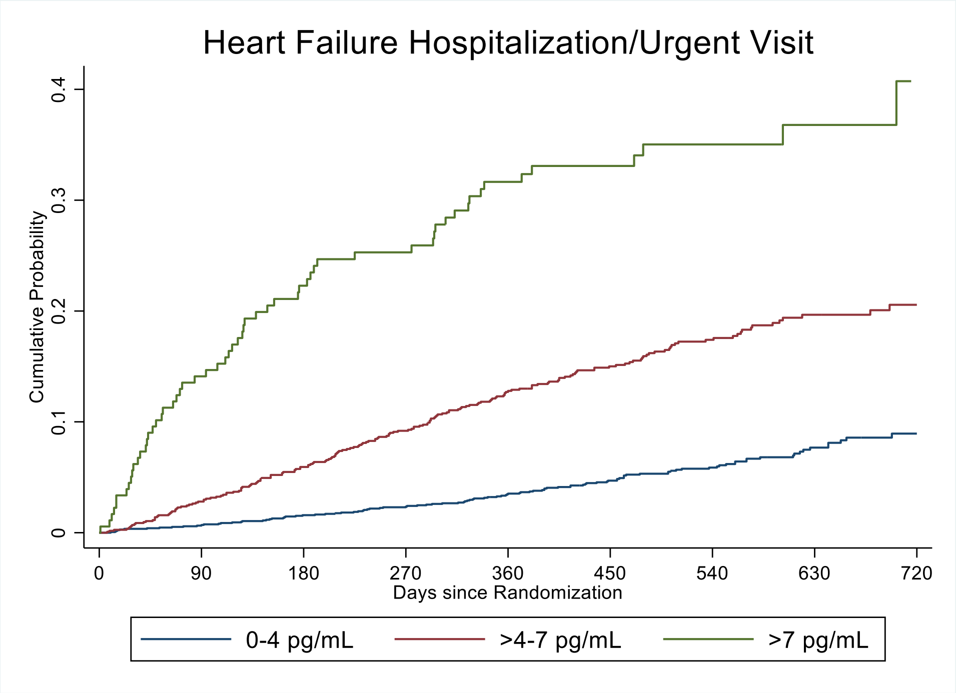

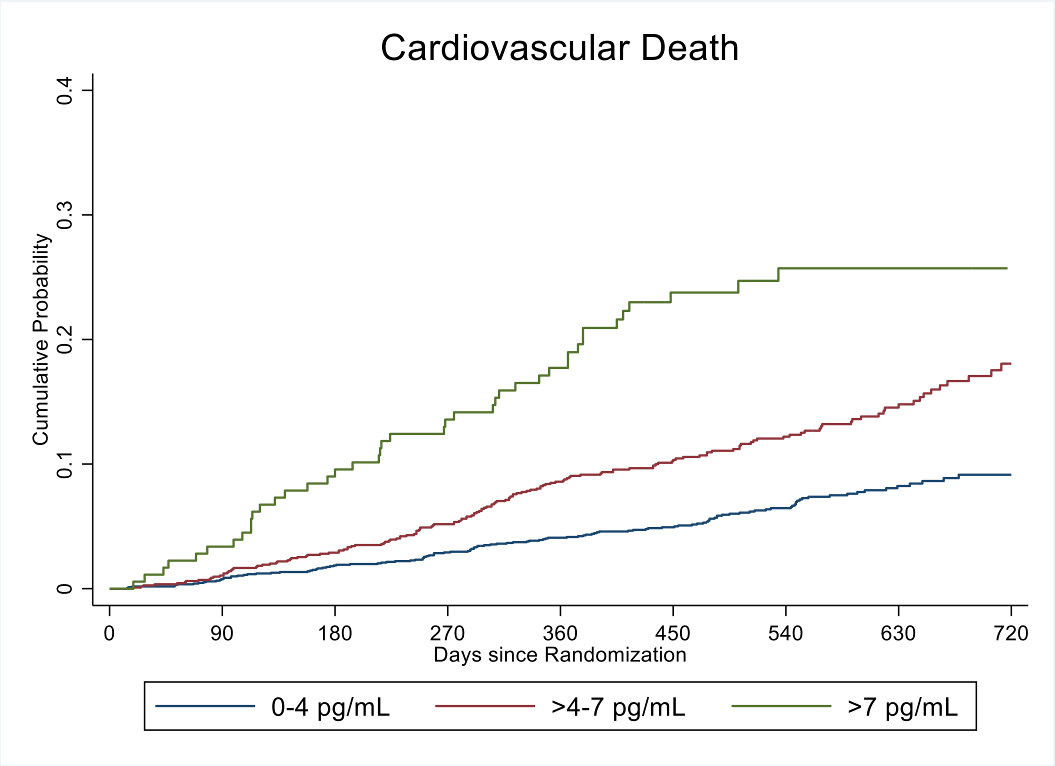
Cardiovascular Death
Cumulative Probability
0
0.1
0.2
0.3
0.4
0
90
180
270
360
450
540
630
720
Days since Randomization
0-4 pg/mL
>4-7 pg/mL
>7 pg/mL

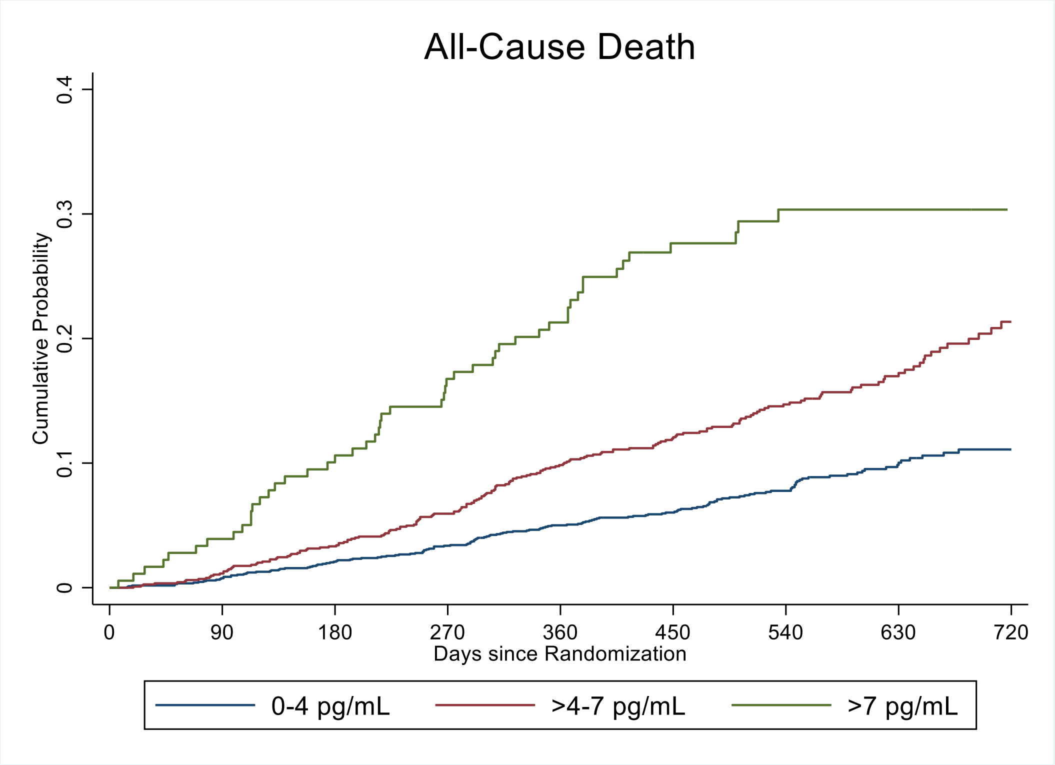
All-Cause Death
Cumulative Probability
0
0.1
0.2
0.3
0.4
0
90
180
270
360
450
540
630
720
Days since Randomization
0-4 pg/mL
>4-7 pg/mL
>7 pg/mL

Supplementary Figure 3: Kaplan-Meier curves showing the effect of treatment group on the primary outcome according to ET-1 tertile

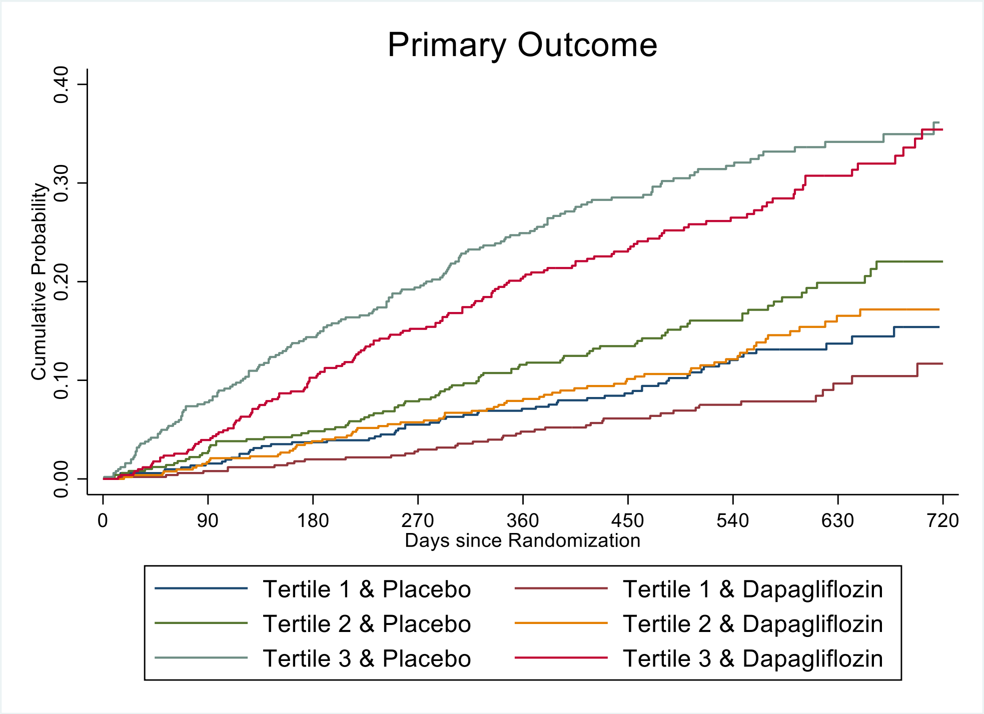

Supplementary Figure 4: Effect of dapagliflozin (vs. placebo) on change in eGFR from baseline, according to baseline ET-1 tertiles

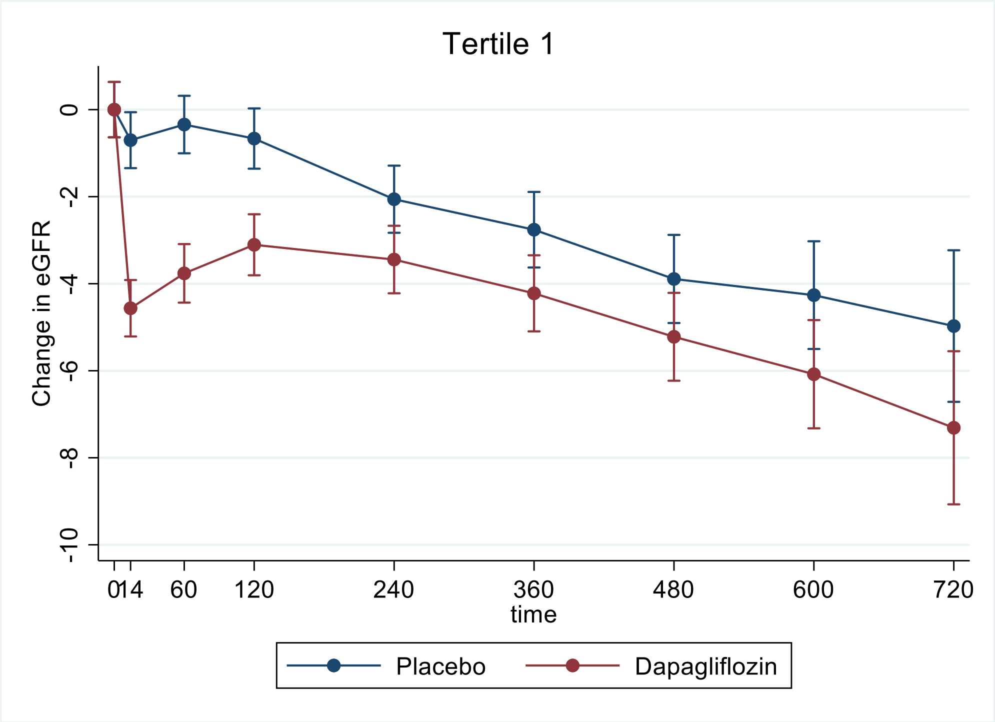

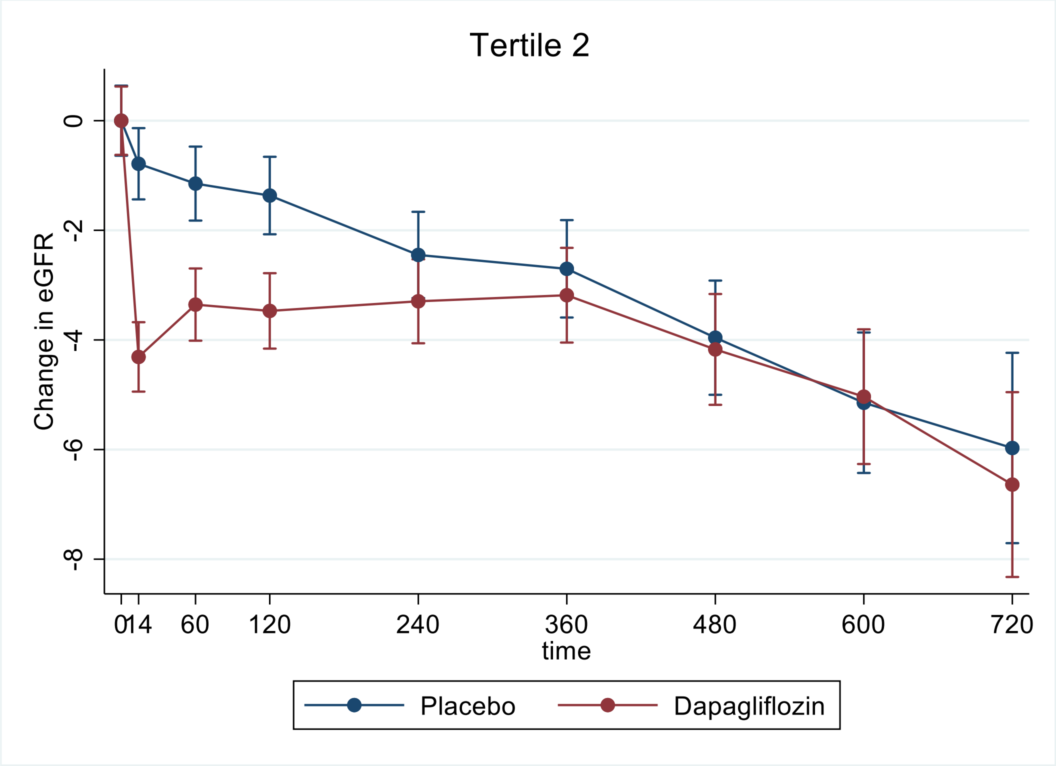

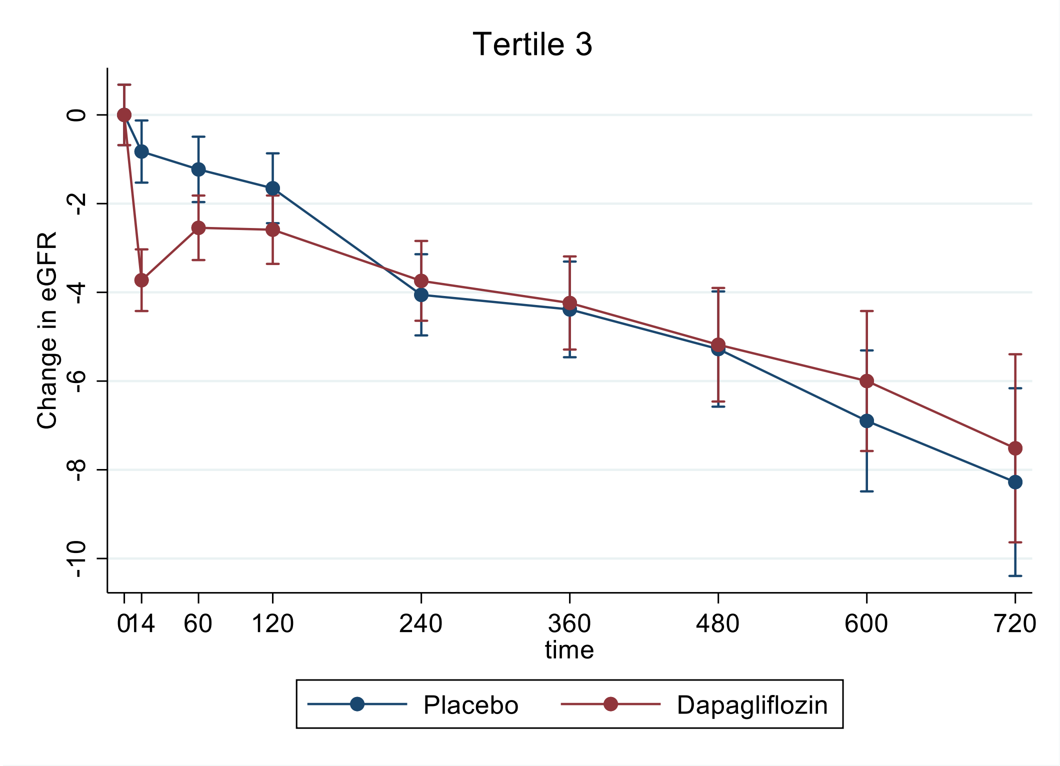
Tertile 3
Change in eGFR
0
-2
-4
-6
-8
-10
0
14
60
120
240
360
480
600
720
time
Placebo
Dapagliflozin

Supplementary Figure 5: Association between change in ET-1 from baseline to 12 months and subsequent risk of the primary outcome

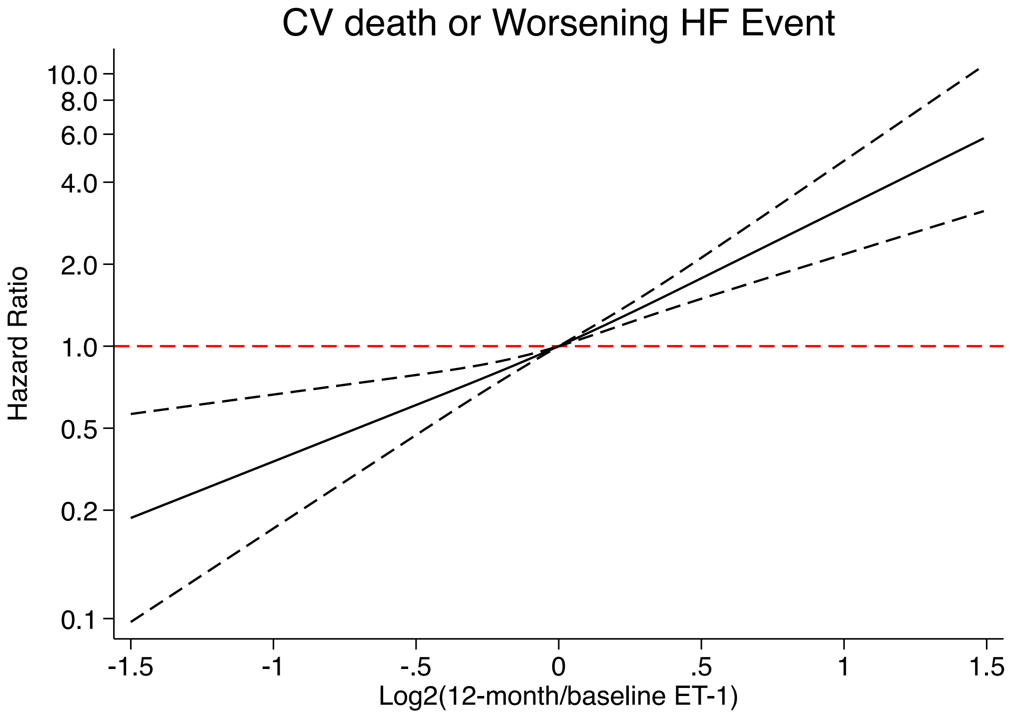

This figure displays the subsequent risk of the primary composite outcome according to the change in ET-1 concentration from baseline to 12 months. HR for the primary outcome according to the log2-transformed ratio of 12 months to baseline ET-1 were modelled using restricted cubic spline analysis adjusted for log-transformed baseline ET-1, randomised treatment, history of heart failure hospitalization and stratified by diabetes status. The referent point is patients with no change in ET-1. The dotted lines represent 95% CIs of the HR estimates. A value of 1.0 and -1.0 on the X-axis represents a doubling and halving in ET-1 from baseline to 12 months, respectively.

SUPPLEMENTAL TABLES

Supplementary Table 1: Event rate (per 100 person-years) and hazard ratios for trial outcomes according to baseline ET-1 group

Supplementary Table 2: Adverse events related to randomized therapy, according to baseline ET-1 tertile

Supplementary Table 1: Event rate (per 100 person-years) and hazard ratios for trial outcomes according to baseline ET-1 group

	Group 1<br>(0-4 pg/mL)<br>N=1,724	Group 2<br>(>4-7 pg/mL)<br>N=1,145	Group 3<br>(>7 pg/mL)<br>N=179	*P-Trend*
**Primary Endpoint (Worsening HF or cardiovascular death) – no. (%)**	202 (11.7)	280 (24.5)	79 (44.1)	<0.001
- **Event rate per 100 person-years (95% CI)**	8.0 (7.0-9.2)	18.5 (16.5-20.8)	42.6 (34.2-53.1)	
- **Unadjusted HR (95% CI)**	1.00 (ref)	2.26 (1.88-2.71)	5.08 (3.91-6.60)	
- **Adjusted HR (95% CI)***	1.00 (ref)	1.61 (1.32-1.95)	2.44 (1.79-3.32)	
- **Adjusted HR (95% CI)†**	1.00 (ref)	1.51 (1.24-1.83)	2.28 (1.68-3.11)	
**Hospitalization or urgent visit for HF - no. (%)**	109 (6.3)	191 (16.7)	60 (33.5)	<0.001
- **Event rate per 100 person-years (95% CI)**	4.3 (3.6-5.2)	12.6 (11.0-14.5)	32.4 (25.1-41.7)	
- **Unadjusted HR (95% CI)**	1.00 (ref)	2.87 (2.27-3.64)	7.10 (5.17-9.76)	
- **Adjusted HR (95% CI)***	1.00 (ref)	1.98 (1.54-2.55)	3.41 (2.33-4.99)	
- **Adjusted HR (95% CI)†**	1.00 (ref)	1.88 (1.45-2.42)	3.24 (2.22-4.75)	
**Cardiovascular death – no. (%)**	121 (7.0)	152 (13.3)	42 (23.5)	<0.001
- **Event rate per 100 person-years (95% CI)**	4.7 (3.9-5.6)	9.3 (7.9-10.9)	18.3 (13.5-24.7)	
- **Unadjusted HR (95% CI)**	1.00 (ref)	1.91 (1.50-2.43)	3.74 (2.63-5.32)	
- **Adjusted HR (95% CI)***	1.00 (ref)	1.33 (1.03-1.72)	1.46 (0.97-2.21)	
- **Adjusted HR (95% CI)†**	1.00 (ref)	1.22 (0.94-1.59)	1.34 (0.88-2.02)	
**All-cause mortality – no. (%)**	148 (8.6)	182 (15.9)	51 (28.5)	<0.001
- **Event rate per 100 person-years (95% CI)**	5.7 (4.9-6.7)	11.1 (9.6-12.8)	22.2 (16.9-29.2)	
- **Unadjusted HR (95% CI)**	1.00 (ref)	1.88 (1.51-2.34)	3.74 (2.72-5.15)	
- **Adjusted HR (95% CI)***	1.00 (ref)	1.37 (1.09-1.73)	1.71 (1.18-2.48)	
- **Adjusted HR (95% CI)†**	1.00 (ref)	1.28 (1.01-1.62)	1.57 (1.08-2.28)	

*Models for death/hospitalization outcomes adjusted for age, sex, treatment arm, race, region, duration of heart failure, previous heart failure hospitalization, heart rate, systolic blood pressure, body mass index, New York Heart Association classification, left ventricular ejection fraction, estimated glomerular filtration rate, etiology of heart failure, history of atrial fibrillation, diabetes and NT-proBNP.
† Model adjusted as for model * with additional adjustment for baseline high-sensitivity Troponin T

Heart failure (HF), confidence interval (CI), hazard ratio (HR)

Supplementary Table 2: Adverse events related to randomized therapy, according to baseline ET-1 tertile

	Tertile 1		Tertile 2		Tertile 3	
	**Dapa**	**Placebo**	**Dapa**	**Placebo**	**Dapa**	**Placebo**
**Any discontinuation – no. (%)**	57 (11.3)	32 (6.3)	52 (9.9)	63 (12.7)	61 (12.1)	70 (13.9)
**Discontinuation due to AE – no. (%)**	27 (5.4)	13 (2.5)	18 (3.4)	28 (5.6)	29 (5.7)	35 (7.0)
**Adverse events – no. (%)**						
Volume depletion	26 (5.2)	36 (7.1)	42 (8.0)	26 (5.2)	46 (9.1)	40 (8.0)
Renal	17 (3.4)	22 (4.3)	35 (6.7)	28 (5.6)	34 (6.7)	45 (9.0)
Fracture	10 (2.0)	12 (2.4)	10 (1.9)	8 (1.6)	17 (3.4)	16 (3.2)
Amputation	2 (0.4)	4 (0.8)	5 (1.0)	0 (0)	3 (0.6)	4 (0.8)
Major hypoglycemia	0 (0)	2 (0.4)	1 (0.2)	0 (0)	1 (0.2)	0 (0)

The safety analysis included only patients who took at least one dose of randomized treatment and patients with baseline ET-1 (3046 patients). AE = adverse event Dapa = dapagliflozin.
